# Evaluation of anterior cruciate ligament surgical reconstruction through finite element analysis

**DOI:** 10.1038/s41598-022-11601-1

**Published:** 2022-05-16

**Authors:** Konstantinos Risvas, Dimitar Stanev, Lefteris Benos, Konstantinos Filip, Dimitrios Tsaopoulos, Konstantinos Moustakas

**Affiliations:** 1 Department of Electrical and Computer Engineering, University of Patras, 25500 Patras, Greece; 2 Institute of Bioengineering, École Polytechnique Fédérale de Lausanne, 1015 Lausanne, Switzerland; 3 Institute for Bio-Economy and Agri-Technology (IBO), Centre of Research and Technology-Hellas (CERTH), 6th km Charilaou-Thermi Rd, 57001 Thessaloniki, GR Greece

**Keywords:** Biomedical engineering, Mechanical engineering

## Abstract

Anterior cruciate ligament (ACL) tear is one of the most common knee injuries. The ACL reconstruction surgery aims to restore healthy knee function by replacing the injured ligament with a graft. Proper selection of the optimal surgery parameters is a complex task. To this end, we developed an automated modeling framework that accepts subject-specific geometries and produces finite element knee models incorporating different surgical techniques. Initially, we developed a reference model of the intact knee, validated with data provided by the Open Knee(s) project. This helped us evaluate the effectiveness of estimating ligament stiffness directly from MRI. Next, we performed a plethora of “what-if” simulations, comparing responses with the reference model. We found that (a) increasing graft pretension and radius reduces relative knee displacement, (b) the correlation of graft radius and tension should not be neglected, (c) graft fixation angle of 20$^{\circ }$ can reduce knee laxity, and (d) single-versus double-bundle techniques demonstrate comparable performance in restraining knee translation. In most cases, these findings confirm reported values from comparative clinical studies. The numerical models are made publicly available, allowing for experimental reuse and lowering the barriers for meta-studies. The modeling approach proposed here can complement orthopedic surgeons in their decision-making.

## Introduction

The anterior cruciate ligament (ACL) rupture is a prevalent knee injury during sports activities, especially those involving abrupt pivoting in conjunction with high tissue loading^1,2^. Usually, people who undergo this type of injury experience knee instability and are more prone to develop osteoarthritis, which can potentially lead to obesity, diabetes, loss of mobility, and life quality deterioration^3^. Remarkably, ACL demonstrates a poor healing capacity that eradicates in complete rupture^4^. In this case, the total replacement of the native tissue with a graft is required as treatment. The graft can be either biological (autograft or allograft depending on its origin, namely from the patient itself or a donor) or synthetic, and it is fixed through tunnels that are drilled through the femur and tibia bones^5^. This procedure is known as anterior cruciate ligament reconstruction (ACLR), and features different methodological approaches regarding the surgical techniques and graft properties^6,7^. The surgical approaches can be classified based on the orientation of tunnel drilling, such as the anteromedial (AM) and transtibial (TT) portal techniques^8–10^. The number of tunnels on each bone is another example of categorizing ACLR methods, with the single bundle (SB) and double bundle (DB) being the most prominent^11–13^. The objective of the latter method is to reconstruct both the AM and posterolateral (PL) bundles to effectively resemble the anatomy and restore the kinematics of the native ACL^14^. Moreover, graft properties, such as radius, pretension, and harvesting site, are important factors that can potentially affect the results of ACLR^11,15^. The in vivo evaluation of the post-surgery knee functionality and behavior is a demanding task due to the nature of these experiments, which introduce high costs, increased time consumption, and technical challenges^16^. To this end, finite element (FE) analysis has emerged as an alternative assessment tool at the disposal of biomedical researchers and healthcare professionals. FE analysis is a valuable modeling and simulation approach of computational biomechanics that exhibits great potential in creating models with high standards of validity. An advanced numerical model can be utilized to evaluate the combined effect of various ACLR parameters, offering the ability to reproduce surgical scenarios that otherwise would require a significantly high number of patients and the arrangement of complex experimental setups. It can also be utilized in “what-if” scenarios to study the response of the underlying soft tissues in different movement and loading conditions in a pre-clinical setting.

One of the vital advantages of FE analysis is that it offers subject-specific modeling of the knee joint complex. This can be accomplished through segmentation and 3D reconstruction of magnetic resonance imaging (MRI) and computational tomography (CT) images to acquire models that feature the subject’s geometric characteristics^17,18^. In the case of ACLR this is crucial since the anatomical footprint of the native tissue serves as an indicator for positioning the bone tunnels^10,19,20^. Hence, antecedent knowledge of the subject-specific bone geometric characteristics could be pivotal to surgery planning. Many FE studies focused on comparing ACLR methods to identify the effect of tunnel number and placement on tissue response and knee kinematics restoration^21–32^. Moreover, the material properties assigned to each reconstructed anatomical structure are of great importance to capture their biomechanical behavior. Several FE studies manipulated MRI data in an attempt to adjust these parameters and accurately model the subject-specific features of the menisci, cartilages, and ligaments^33–36^. Regarding ligaments, a common approach in previous FE studies was to model them as springs that demonstrate a pure tensile, nonlinear stress-strain behavior^36–41^. However, the stiffness and prestrain values assigned to these constitutive models were adopted from the literature. Thus, subject-specific characteristics were either disregarded or predicted by performing sensitivity analyses and compared with experimental data^36,40^. The same approach was considered for graft modeling^26,38,41^, although 3D graft models were the most common approach in relevant FE analyses^23,24,30,31,42–48^. The graft material properties were adapted to represent distinct harvesting sites^30,31,42,43,46,48^.

As far as the validation is concerned, many studies made use of joint mechanics experimental data collected from in vivo or in vitro experiments^26,38,41,49^. Also, results of previous studies that utilized validated FE models have been used as a benchmark for subsequent researches^23,30,42,44^. A usual approach was to develop a baseline model representing the healthy knee with verified kinematic response to various loading conditions. Subsequently, boundary conditions that simulate clinical exams, such as the anterior drawer test^24,27,31,38,41–44,46,47^, or dynamic movements, such as gait^26,27^, were applied to the numerical model. Therefore, the ACLR simulation results were compared with the reference model (RM) output to attain insight into the effect of the investigated surgery parameters^22,24,27,38,42,45,47^.

In this work, we proposed a computational biomechanics pipeline that incorporates all key steps of ACLR surgery modeling. The aim of our study was twofold: (a) enabling realistic subject-specific modeling of all the parameters and aspects of the real-life ACLR, such as the applied surgery technique and the geometric graft characteristics, material properties, pretension, and placement through the bone tunnels, and (b) assembling FE models of the knee joint that can be used to evaluate these constitutive modeled parameters in a Lachman test simulation. Subject-specific modeling is achieved through MRI data manipulation. The entire pipeline is automated through Python scripting and is materialized by employing open-source software tools. The graphical interfaces of each dedicated software are used only for inspecting the generated models. This minimizes the time and effort of ACLR modeling and FE model generation that could be beneficial for sensitivity studies. Furthermore, we proposed an approach for estimating stiffness directly from MRI data that can be easily incorporated in an automated workflow, such as the one presented here. To the best of our knowledge, the correlation between graft pretension and graft radius was not thoroughly investigated before. Therefore, we examined in detail this aspect of ACLR. The results are comparable to previous related FE and clinical studies. This provides added value to the impact of the proposed workflow that could be deployed pre-surgery as a decision support tool for ACLR planning and preparation. Finally, the numerical models developed in this study are made publicly available for research transparency, allowing for reuse and reproducibility.

## Methods

**Figure 1:**
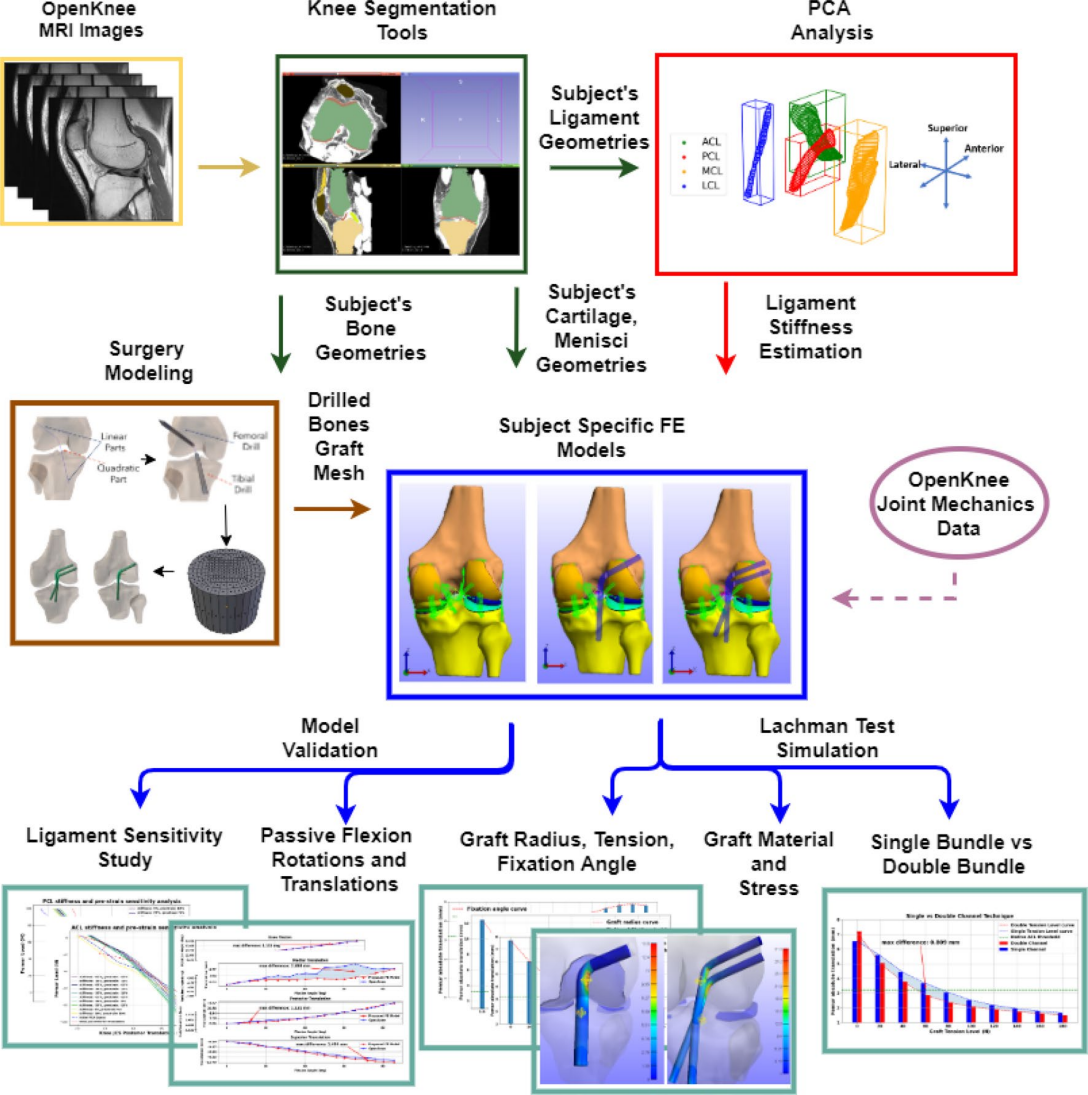
Overview of the proposed workflow. MRI data are used to propose a surgery plan for subject-specific ACLR. First, we use open-source segmentation tools to acquire geometries of the anatomical structures. Next, PCA is applied to ligament geometries to acquire subject-specific estimation of stiffness (PCA block). The bone geometries are used in the Surgery Modeling tool to drill the tunnels. During this process, the graft mesh is also generated. Subsequently, we automatically generate FE models of the knee joint. Joint mechanics data from the open-source Open Knee(s) project are used to develop and validate a RM. Then, the surgery parameters are evaluated by performing a FE simulation of the Lachman clinical examination. The performance of models that correspond to the ACL reconstructed knee joint are compared to that of the RM. The measurements of interest are relative knee displacement and graft maximum principal stress (Diagrams.net, v17.2.1, https://www.diagrams.net/).

An overview of the proposed workflow is presented in Fig. 1. In brief, the current pipeline utilizes subject-specific geometries that are generated by segmented MRI images of the knee joint, acquired from the open-source Open Knee(s) project^50^. The meshes of the anatomical structures are acquired using open-source knee segmentation tools^51^. The bone geometries are the input data to the surgery modeling workflow, which relies on the Blender software^52^ to model the primary steps of ACLR and generate the graft geometry. In addition, we perform principal component analysis (PCA) on the ACL, posterior cruciate ligament (PCL), medial collateral ligament (MCL) and lateral collateral ligament (LCL) geometries to estimate their mean cross-sectional area and provide a first estimation of each ligament’s stiffness. Subsequently, the FE knee models are generated based on the selected ACLR parameters and material properties. Finally, validation experiments and “what-if” simulations of the Lachman test are performed to gain an insight into different aspects of ACLR.

### ACL reconstruction surgery modeling

**Figure 2:**
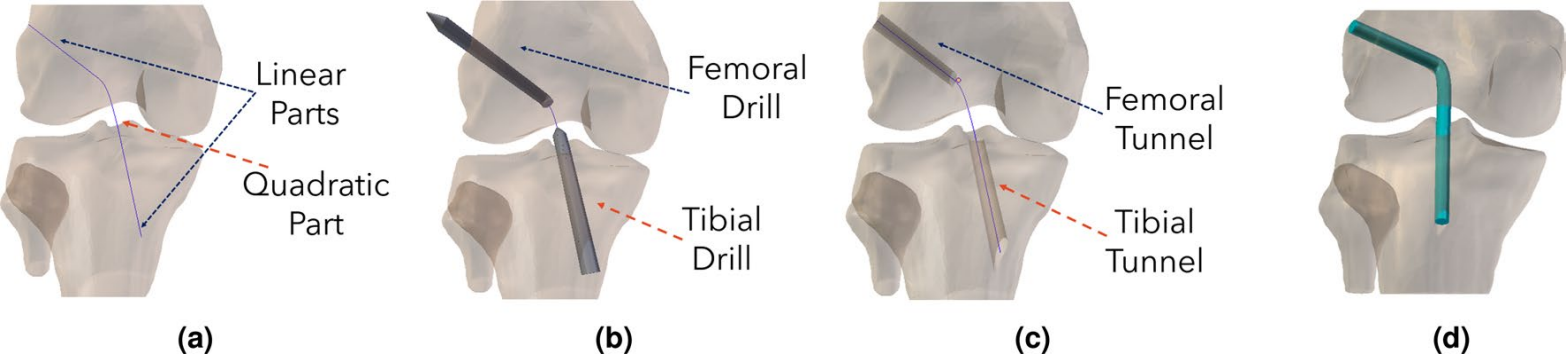
Overview of ACLR surgery modeling workflow. The bone tunnels are “drilled” using cylindrical objects that trace a NURBS curve. (**a**) The NURBS curve is defined based on the anatomical landmarks, (**b**) cylindrical meshes are used for the tunnel “drilling” procedure, (**c**) the “drilled” tibia and femoral tunnels, and (**d**) the graft is morphed to the path and placed precisely through the anatomical landmarks.

The workflow begins with the ACLR modeling framework, where the key surgery parameters are determined. The first step is to choose between SB or DB reconstruction techniques to decide the number of the tunnels. The SB technique refers to a single tunnel through both bone surfaces, whereas the DB requires two tunnels through each bone. In the case of SB reconstruction, two landmarks are selected on both the tibia and femur bone surfaces, representing the onset and exit points of the tibial and femoral tunnels. Thus, a total of four points are used to define a nonuniform rational B-spline (NURBS) curve. The path is composed of two line segments within the bones and a curved section that covers the intermediate space between them, as illustrated in Fig. 2a. The trajectory of the curve is used to “drill” the tunnels and properly place the graft. The tunnels are “drilled” using round-shaped cylinder objects with a user-specified radius that trace the trajectory. A Boolean operator is applied to remove the intersection between the cylinder and the bones’ mesh. These steps are illustrated in Fig. 2b,c, respectively.

Subsequently, the workflow generates the graft mesh based on the given landmarks, radius, and path, as seen in Fig. 2d. The user can define the mesh type (hexahedral or tetrahedral), element density, and the number of graft bundles. Starting from a grid mesh, we form cylindrical meshes consisting of brick-like elements stacked together to form the final graft mesh. The length of each cylinder affects the mesh density; this offers the advantage to generate a denser mesh in areas of interest, such as the graft insertion to the femoral tunnel, and therefore, to increase the precision of the numerical solution in subsequent FE analyses. Moreover, a single graft can consist of multiple bundles. Towards this direction, copies of the graft are created and placed symmetrically to fill the space of a circular area with a radius equal to the selected graft radius. Then, the bundle meshes are twisted together to shape a rope-like object. In this fashion, the graft mesh generation is completed, and the final graft mesh is attached to the curve and placed accurately through the tunnels and the selected landmarks. When a DB surgery approach is desired, the entire process is repeated for the second graft.

Blender only supports exporting of polygon meshes (explicit surface representation), while the subsequent FE modeling requires volumetric meshes of the structures where internal stresses are estimated. In this context, we utilized the Blendbridge^53^ software to convert the graft surface mesh to a hexahedral volumetric mesh. Furthermore, tetrahedral meshing is also supported using the TetGen software^54^. Additionally, to improve mesh quality, we use the Gmsh^55^ software and, in particular, the 3D Frontal unstructured algorithm for refining the hexahedral mesh. Finally, we performed a mesh quality test using the meshing quality filters of Paraview software^56^. Along these lines, it should be stressed that all the above steps are executed through scripting. More technical details about the surgery modeling step and graft mesh quality metrics are provided in the supplementary materials (Sect. 1).

### Finite element model development

**Figure 3:**
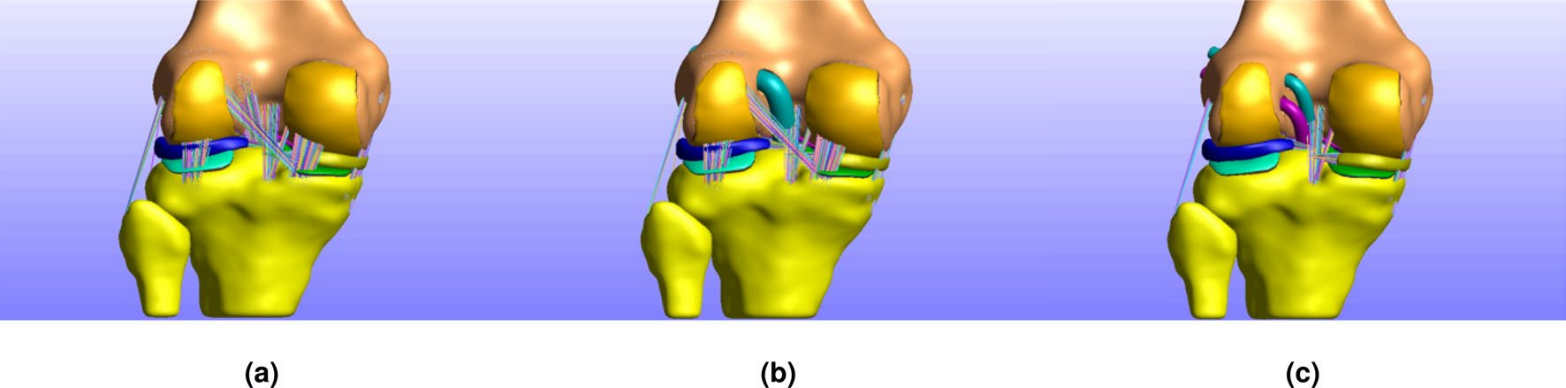
FE model versions. In this work, we developed three different versions of a FE knee model that correspond to (**a**) the healthy knee used for reference and comparison (RM), (**b**) the ACL reconstructed knee with the SB approach, and (**c**) the DB technique.

Within the confines of this research study, we developed three different FE models using the FEBio Software Suite^57^. FEBio is an open-source FE software tool specially designed for biomechanics, capable of solving nonlinear large deformation problems. To automate and precipitate the model assembly process, all the steps that would have been performed using the FEBio interface are executed through scripting. All three models resemble the same subject and correspond to healthy (Fig. 3a) and ACL reconstructed knee, exploring different surgery options such as the SB and DB techniques mentioned above and depicted in Fig. 3b,c respectively. We validated the response of the healthy knee model and used it as the RM to compare with the models that correspond to the ACLR surgery.

#### Geometries

The surface meshes of the femur, tibia, and fibula bones are used directly from the Open Knee(s) project database. The tibial and femoral cartilages and the menisci volumetric meshes are generated using open-source knee segmentation tools^51^. Finally, the graft meshes are generated by the surgery modeling approach discussed above. More details about mesh properties can be found in the supplementary materials (Sect. 2.1).

#### Material properties

Efficient material models, which can describe the mechanical response of the knee joint tissues, are of significant importance. In the present FE model, the bones are modeled as rigid bodies since their deformation is negligible compared to other present anatomical structures^21,28,31,38^. The focus of our study was to examine parameters of ACLR surgery without considering bone deformation due to bone - graft contact. Therefore, we decided to model the rigid bones as triangular surface meshes^48^. This offers the double advantage of enabling rigid body kinematics and constraints while significantly reducing the complexity of the problem. The femoral and tibial cartilages are partitioned into three layers to represent their anatomical structure^58,59^. Each layer is modeled as a hyperelastic, uncoupled Mooney-Rivlin material^60^. This type of material is considered to be sufficient enough for modeling the nonlinear nature of incompressible materials^61^. In addition, the menisci are modeled using the orthotropic Fung elasticity model^62^. The material properties of each anatomical structure are presented in more detail in the supplementary materials (Sect. 2.2).

**Figure 4:**
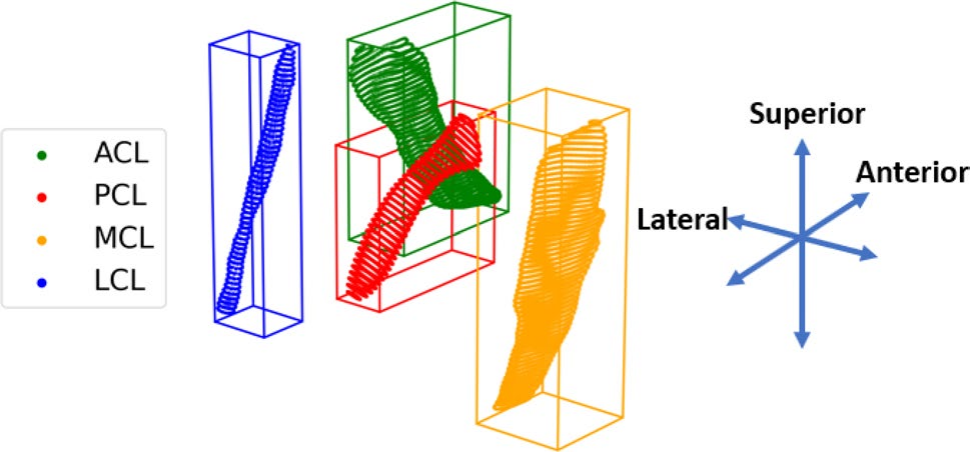
Estimating ligament stiffness from MRI. Cross-sections and the bounding box of each ligament surface mesh are presented. Multiple cross-sections are taken along the principal ligament axis derived by PCA. The ligament’s cross-sectional area is estimated as the mean of the areas of each slice.

The ligaments are modeled as discrete elements established between two sets of vertices that lie on the bone surface meshes and represent each ligament’s origin and insertion site. The ACL, PCL, MCL and LCL are modeled as bundles of nonlinear springs, with a characteristic stress-strain curve that is shaped after equation (1), proposed by^37^:

1 $$\begin{aligned} F = {\left\{ \begin{array}{ll} 0 &{} \varepsilon < 0 \\ \frac{1}{4}k\varepsilon ^2/ \varepsilon _l &{} 0\le \varepsilon \le 2\varepsilon _l \\ k(\varepsilon - \varepsilon _l) &{} \varepsilon > 2\varepsilon _l \end{array}\right. } \end{aligned}$$

where parameter *k* is the ligament stiffness, $\varepsilon _l$ the linear strain limit that has a default value of 0.03, and $\varepsilon$ the strain of the ligament derived from its current length and the zero slack length $L_0$. The $L_0$ parameter is computed as $L_0 = L_r/(1+\varepsilon _r)$, where $L_r$ is the reference spring length, equal to the spring length at the beginning of the simulation, and $\varepsilon _r$ is the reference strain parameter. The stiffness parameter *k* has units of Newtons and is derived as $k = E \cdot A$, where *E* is the ligament’s Young’s modulus in *MPa*, and *A* is the ligament’s cross-sectional area in ${mm}^2$. The estimated stiffness value is distributed evenly to each spring by dividing *k* by the number of ligament springs. To achieve subject-specific ligament modeling, we estimated the mean cross-section area of each ligament from the segmented MRI data. Specifically, we used PCA to find the principal axis of each mesh. This axis was defined as the normal vector of consecutive planes with an offset of one unit for a range equal to the length of the main diagonal of each mesh bounding box, as illustrated in Fig. 4. Each plane corresponds to a slice of the mesh, and for each slice, the area was estimated. Therefore, for each ligament, the mean cross-sectional area was the average area of these slices. The Young’s modulus values are derived from literature^63,64^. The estimated stiffness and reference prestrain values are used as an initial guess for a sensitivity analysis where the objective is to discern the optimal combination of these parameters. The results will be presented in the relevant “Results” section.

Regarding the menisci roots, they are modeled as bundles of linear tension-only springs with a total stiffness value of 2000 N/mm equally distributed to each spring^33,65^. Moreover, we modeled the posterior capsule, anterolateral and arcuate ligaments. These were included after the model validation simulations since the model was also used to experiment with dynamic movements where the role of these secondary ligaments cannot be ignored^66–68^. The values and constitutive models^39,69–72^ for the above soft tissues are presented in the supplementary materials (Sect. 2.3).

The grafts are modeled with a transversely isotropic Mooney-Rivlin material. It appears to be an appropriate constitutive model for ligaments, tendons, and muscles since it was developed for modeling soft tissues^73^. To successfully represent different tissues that serve as graft harvesting sites, we explored three different versions of the material with values corresponding to the semitendinosus, gracilis, and patellar tendon^42,43^.

#### Contact modeling

An essential step in the FE model construction workflow is to properly select the contact surfaces of the different materials and apply suitable contact models. First, we utilize rigid contacts to attach deformable compartments to rigid bodies. Subsequently, we define the surface pairs that feature a sliding elastic contact^74^ to prevent penetration and to consider knee joint articulation kinematics and load transmission^58,59,75,76^. Sliding elastic contact is also used to model the interaction between the graft and the bones. Finally, a tied contact is applied to connect the top part of the graft surface to the surface of the femoral tunnel. An overview of the formed contact pairs is presented in the supplementary materials (Sect. 3).

#### Knee joint coordinate system

The knee joint coordinate system is defined according to the method proposed by Grood and Suntay^77^. It is modeled as a four-link kinematic chain, where each joint is a cylindrical pair that allows only relative rotation and translation between the links that define it. We followed this approach to carry out a direct comparison of the developed FE model with cadaver joint kinematics and kinetics provided by the Open Knee(s) project^50^. In addition, the femur and tibia coordinate systems were determined based on the methodology provided by Erdemir^50^ and described in more detail in the supplementary materials (Sect. 3.4).

#### Boundary conditions

The structural mechanics analysis in FEBio was used to perform the different simulation scenarios. First, we reproduced the anterior and posterior drawer tests described in the Open Knee(s) project. These experiments were used as the baseline for the ligament parameters sensitivity analysis. In this case, initial knee flexion was prescribed up to the respective value. Then, the anterior/posterior force was prescribed at the origin of the femoral coordinate system with a ramp function for the duration of a subsequent simulation step. Moreover, we carried out a passive flexion simulation to validate the model’s behavior and investigate if the selected parameters can produce an approximately similar behavior compared to the Open Knee(s) ground truth data. As passive flexion, we consider the knee motion where the relative motion between femur and tibia is restrained only by the present anatomical structures when a flexion angle is prescribed about the medial-lateral knee axis. Hence, the flexion angle was prescribed with a ramp function for a single simulation step. During these validation simulations, the tibia was constrained in all degree of freedoms (DoFs) while the femur was unconstrained. The validated FE model was presented in Fig. 3a. It epitomizes the healthy knee and is used as the RM to obtain a benchmark behavior for comparison with “what-if” models.

Additionally, we performed simulations that aim to reproduce the Lachman test, one of the most common passive orthopedic ACL examinations in the case of a suspected ACL rupture. It assesses the anterior displacement of the tibia as the ACL primary function is to restrain it. The patient lies in a supine position, and the knee is flexed at about 20$^\circ$-30$^\circ$. The examiner stabilizes the femur’s distal end with one hand and applies an anteriorly directed load on the proximal tibia^16^. The test is performed on both the healthy and the potentially injured leg in order to acquire a ground truth feel, or measurement^78–80^. We reproduced this scenario to assess different parameters of the ACL surgery, based on their efficiency to minimize relative knee displacement along the axis of the direction of force action, similar to other studies^24,38,42–44,46^. To eliminate ambiguity, we clarify that relative displacement refers to the posterior femoral translation analogous to anterior tibia displacement. We examined the SB and DB models illustrated in Fig. 3b,c and compared their response with the RM benchmark. The SB model was used to investigate graft pretension, radius, and material properties. It was also used to evaluate knee flexion during graft fixation. The DB model was used in a comparison study between the SB and DB approaches.

The boundary conditions for the Lachman test are applied in two steps: (1) *Graft pretension step*. Initially, the knee is flexed up to the desired fixation angle. Then, a pressure load is distributed on the graft bottom surface equal to the desired pretension force divided by the graft cross-section area. The graft top part is tied to the femur. At the end of the pretension step, the graft bottom surface is fixed in all DoFs, and the knee returns to the default full extension pose. This step is omitted for the RM Lachman simulation and is applied only to the ACLR FE knee models. (2) *Lachman Manoeuvre step*. The knee is flexed up to 30$^\circ$ and a posterior force of 134 Newtons is applied to the origin of the femoral coordinate system. The tibia is fixed throughout all Lachman simulations, whereas the femur is now fixed only for the internal-external rotational DoF. Since we focus exclusively on studying the relative displacement along the anterior/posterior direction, the role of ACL as a secondary knee rotation stabilizer is not considered. At the end of each simulation, the induced relative knee displacement and graft stresses are retrieved. The relative displacement obtained by the RM is used as a threshold to compare the performance of the ACLR FE knee models.

## Results

The first two subsections are concerned with the proposed RM validation. On that account, we used data from cadaver experiments from the Open Knee(s) project^50^. First, the MRI estimated stiffness values were compared with the reference values^63^. Next, we performed a sensitivity analysis to determine the combination of stiffness and prestrain values for the cruciate ligaments. This was accomplished by replicating the experimental anterior-posterior laxity tests. After adjusting the ligament properties, we performed a passive flexion simulation to verify the RM kinematics behavior. The remaining sections are dedicated to evaluating miscellaneous ACLR options, such as surgery technique, graft pretension, radius, material, and knee fixation angle, in a Lachman test simulation. For this, we developed multiple FE models, and their performance was compared to that of the validated RM model.

### Estimation of ligament stiffness through MRI

In this subsection, the estimated ligament parameters from the available MRI data will be presented. In Table 1, the values for the Young’s modulus^63,64^, estimated area from MRI, reference area (literature values^63^), and stiffness values are demonstrated. A good agreement between the estimated and reference values is found for the ACL and MCL ligaments. A discrepancy is observed for the PCL which stems from the deviation in the cross-sectional area. In the case of LCL, the reference value is larger, although information about the ligament’s cross-sectional area was not reported in reference^63^. The plethora of FE models make use of reference values derived from the literature, which might not be in line with the subject-specific characteristics of these tissues. Therefore, ligament parameters could be derived from MRI data, if available, to accommodate for subject-specific modeling. These results presented here will be further analyzed in the “Discussion” section.

**Table 1:** Comparison of ligament parameters between estimated and reference literature values.

Ligament	Young’s modulus (MPa)	Estimated$^{\dagger }$ area (mm)	Reference$^{\ddagger }$ area (mm)	Estimated stiffness (N)	Reference stiffness (N)
ACL	355	35.94	35	12,759	10,000
PCL	304	42.12	60	12,804	20,100
MCL	355	21.11	24	7496	8250
LCL	355	7.2	N/A	2556	6000

The stiffness values are computed as the product of the cross-section area and Young’s modulus.

$^{\dagger }$ The estimated values from MRI data by applying PCA on the segmented geometries.

$^{\ddagger }$ As “reference” we denote the values that are acquired from^63^.

### Ligament parameter sensitivity analysis and validation

To evaluate the optimal combination of ligaments’ stiffness and prestrain, we performed a sensitivity study where we varied the MRI estimated stiffness values and the reference prestrain rates^37^. The objective function was the mean squared error (MSE) of the relative knee displacement between the FE model and the laxity test experiments obtained from the Open Knee(s) project^50^. These experiments include the knee flexion angle and the anterior force applied to the femur center of mass. The stiffness and prestrain values were altered by a factor $\pm 30\%$ and $\pm 15\%$, respectively, with a $5\%$ step. A total of 91 models were developed for evaluating the behavior of each parameters’ combinations. The two collateral ligaments’ parameters were fixed because their action is less relevant for anterior-posterior movement and ACLR. The derived estimation for the ACL and PCL is used to model the cruciate ligament parameters of the RM.

**Figure 5:**
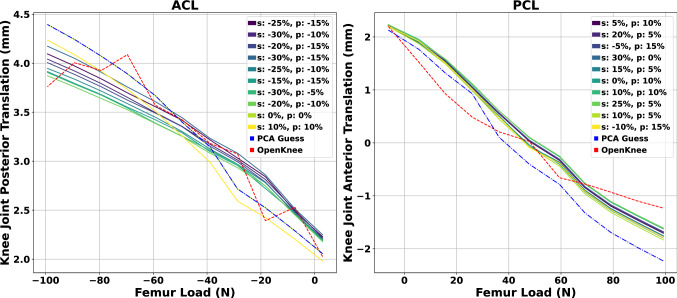
Aggregated results from the sensitivity analysis with the ten best combinations of stiffness and prestrain values for ACL and PCL. The estimated stiffness (PCA method) and recommended prestrain value from Blakenvoort were used as initial guesses. The “s” and “p” denote the stiffness and prestrain percentage change, respectively. In addition, we observed combinations that include the initial stiffness value and changed only the prestrain by a factor.

The aggregated results from the sensitivity analysis with the ten best combinations of stiffness and prestrain values are depicted in Fig. 5. The labels are sorted in ascending order, with the first combination highlighting the minimum MSE as compared to the experimental laxity data. We observed that achieving a good match by varying only the prestrain parameter and keeping the stiffness value unchanged is feasible. Thus, different combinations of stiffness and prestrain values might lead to similar responses. This also highlights the inherent uncertainty of the prestrain factor that affects the overall predicted ligament behavior. Nevertheless, the estimated stiffness leads to model responses that closely resemble the ground truth measurements.

### Passive knee flexion and validation

**Figure 6:**
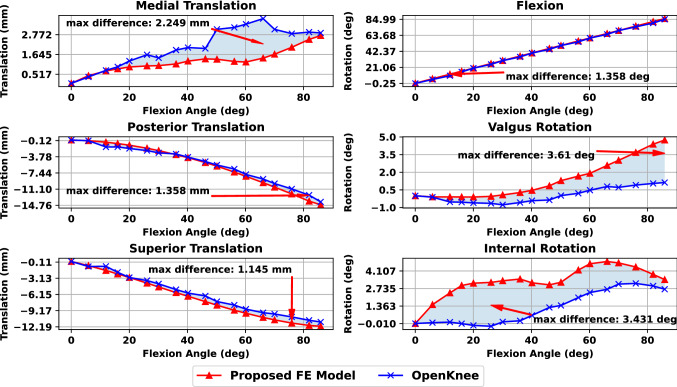
Comparison of simulated and experimental kinematics during passive knee flexion (translations left and rotations right). We observe that the proposed FE model exhibits comparable performance for the translations except for the medial-lateral direction. Regarding rotations, different behavior is evident for the internal-external rotation with a good initial match during varus-valgus rotation.

The Open Knee(s) data set contains experimentally measured passive flexion knee kinematics that we used for model validation. After calibrating ligament parameters based on the previous experiment, we performed a passive knee flexion simulation. We compared the kinematics of the RM model with the ground truth data, as depicted in Fig. 6. We observed that the proposed FE model exhibits reduced medial translation in the range between 20$^\circ$ and 80$^\circ$ with a maximum difference of 2.249 mm. Posterior and superior translations are very similar, with the highest deviations of 1.358 mm and 1.145 mm, respectively. Concurrently, we notice that the knee model exhibits higher valgus rotation, especially after mid-flexion and 40$^\circ$ that attains a peak value of 3.61$^\circ$ at 80$^\circ$ of knee flexion. On the other hand, the internal rotation is higher for the first part of knee flexion with a max difference of 3.431$^\circ$. These differences could be attributed to unmodeled structures such as the patellofemoral joint. Overall, the model exhibits satisfactory kinematics, especially for the primary DoFs involved in the ACLR surgery.

### Effect of graft radius and pretension level

**Figure 7:**
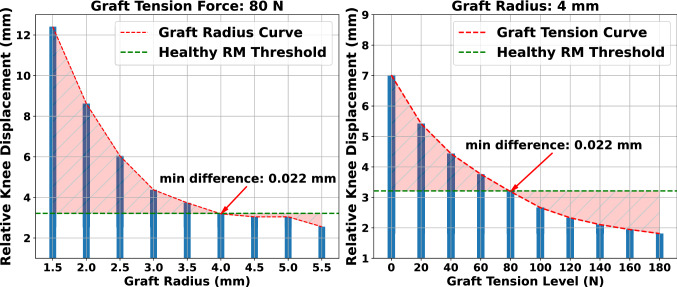
Effect of graft radius and pretension on knee laxity for a semitendinosus graft. The “difference” term refers to the absolute difference in relative knee displacement between each ACLR FE model and the RM. Increasing the graft radius for a specific pretension load reduces relative displacement. The same applies when increasing graft pretension for a fixed value of graft radius.

The graft radius and pretension level are two surgical parameters that influence knee response after surgery. This experiment examined how different combinations of these parameters would influence post-surgery relative knee displacement. We performed a uniform grid sample (2 parameters) but presented it in 2D for clarity, the 80 N graft pretension force, and 4 mm of radius (Fig. 7). The results were compared to those of the RM, which includes the ACL ligament modeled by discrete springs with reference stiffness and prestrain parameters as calibrated with the validation experiments.

In Fig. 7 (left), the effect of the graft radius choice on restraining the relative knee displacement is presented. We observed that an increase in the graft radius limits the relative knee displacement along the force’s line of action. It also appears that knee translation for radii of 4 mm and above is stabilized, as concluded by the graft radius curve slope. In this case, the optimal choice was that of 4.0 mm as highlighted by the filled area between the slope and the RM threshold, entitled as “Healthy RM” in Fig. 7. Moreover, in the right part of Fig. 7, the impact of increasing pretension of the graft before fixation is presented. Again, it is clear that increasing the tensile graft load reduces the relative knee displacement in an approximately exponential fashion. For this simulation, a graft pretension in the range of 60–80 N demonstrates the best performance in achieving a knee displacement proximate to the healthy RM.

### Effect of graft tissue choice

**Figure 8:**
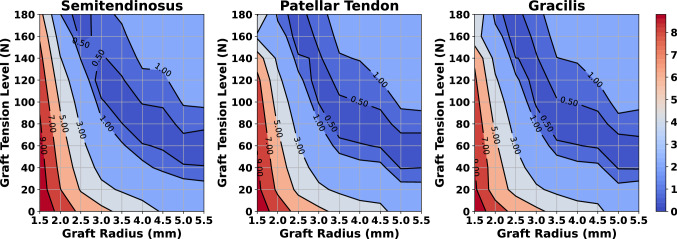
Comparison of three graft materials for different pretension and graft radius values. The absolute difference in relative knee displacement between each ACLR FE model and the RM was annotated using contour lines. Combinations of graft tension and graft radius that are close to the RM are inside the area with an absolute difference below 0.5, highlighted with dark blue color.

It is of particular interest to compare the ability of different graft options to constraint the relative knee displacement after an ACLR surgery. Therefore, we analyzed three different graft materials corresponding to the semitendinosus, patellar tendon, and gracilis harvest sites. Also, we wanted to examine if the tissue response changes its behavior for different combinations of pretension and graft radius levels. In Fig. 8, all combinations of graft pretension and radius values for each of the three grafts are visualized. The simulated displacement was compared with the RM, and the absolute distance was annotated using contour lines. We observed that all three grafts exhibit similar behavior in the range of 60–100 N and radii between 3.5 and 5 mm.

A quantitative description of the performance for the three graft materials is presented in Table 2. There we listed the five best combinations of radius and pretension values that exhibit the lowest MSE compared to the RM. We observed that the most effective combination in terms of minimizing knee laxity is a gracilis graft with a radius of 2.5 mm and a pretension load of 160 N. On the other hand, a small radius requires excessive graft pretension, as observed by this combination and all other pairs with radii 2.5 mm. Moreover, a graft with a 4 mm radius and 80 N pretension demonstrates a consistent performance, as seen with all three materials. In general, grafts with ranges of radius between 3 and 4 mm and pretension between 80 and 120 N perform adequately in restraining relative knee translation.

For each result, we provide the maximum principal stress values. The peak stress was developed around the femoral tunnel insertion area due to the contact between the graft and the internal lateral femoral surface. A noteworthy observation is that a choice of a graft with smaller radii significantly increases stress, as it is evident for the grafts with radii of 2.5 mm. Also, a general inference that can be derived from Table 2 is that stress rises with increasing values of pretension and that grafts with radii around 4 mm can demonstrate a balanced performance for all investigated tissues.

**Table 2:** Qualitative results depicting the five best models and parameter combinations for each of the three grafts.

Semitendinosus				Patellar tendon				Gracilis			
Radius (mm)	Pretension (N)	Error$^{\dagger }$ (mm)	Stress$^{\ddagger }$ (MPa)	Radius (mm)	Pretension (N)	Error (mm)	Stress (MPa)	Radius (mm)	Pretension (N)	Error (mm)	Stress (MPa)
3	120	0.116	31.2	4	80	0.016	23.8	2.5	160	0.012	54.6
4	80	0.122	22.6	2.5	160	0.055	53.7	3.5	80	0.046	33.4
3.5	100	0.186	31.7	3	120	0.067	32.9	4	80	0.061	24.2
5.5	60	0.2	18.6	5.5	60	0.223	22.7	3	120	0.127	33.4
2.5	180	0.257	52.7	3.5	80	0.111	32.7	5.5	60	0.132	23.1

The best performing model (gracilis minimum error) exhibits very high-stress values. A 4 mm radii and 80 N pretension rank well in terms of less stress and satisfying displacement error.

$^{\dagger }$The error is the absolute difference in relative knee displacement between the ACLR FE model and RM.

$^{\ddagger }$ Maximum principal stress.

### Effect of graft fixation angle

**Figure 9:**
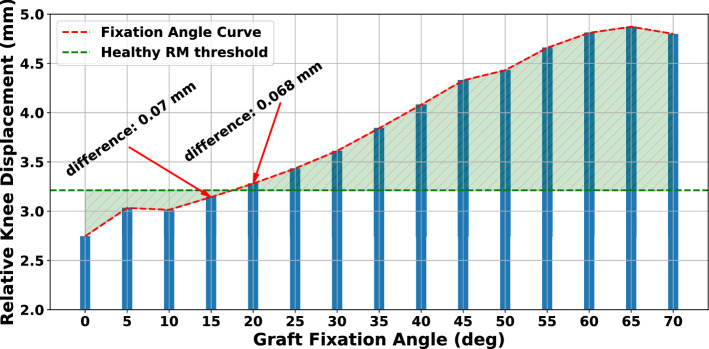
Effect of graft fixation angle on relative knee displacement. The “difference” term refers to the absolute difference in relative knee displacement between the ACLR FE model and the RM. It is noticed that for angles larger than 30$^\circ$ the relative knee laxity increases. In our case, we found the optimal fixation angles are between 15$^\circ$ and 20$^\circ$.

One parameter that conceivably affects the postoperative results of ACLR is the knee flexion angle during graft fixation, with no consensus about the optimal range. In Fig. 9, the results for investigating the influence of graft fixation angle in knee laxity are displayed. We used a semitendinosus graft of 4 mm radius and an applied pretension of 80 N. It is noticeable that increasing the knee flexion angle above 30$^\circ$ when fixing the graft through the bone tunnels leads to an increased relative knee displacement. By inspecting the fixation angle curve, a choice between 15$^\circ$ and 20$^\circ$ produces the closest performance to that of the healthy RM, with an absolute difference in relative knee displacement of approximately 0.07 mm. On the other hand, we observe that for the range between full extension and 10$^\circ$ the knee is overconstrained.

### Single channel versus double channel

**Figure 10:**
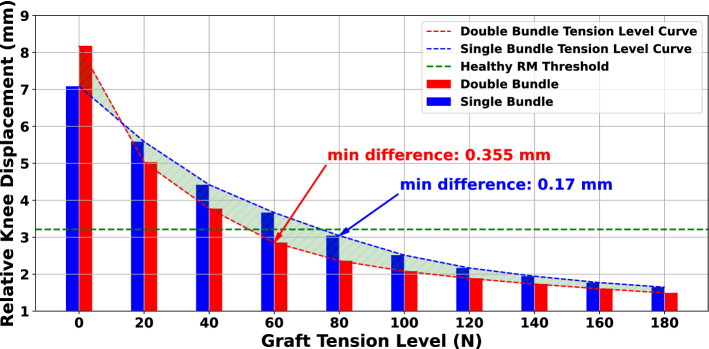
Results for comparing the SB and DB techniques. The “difference” term refers to the absolute difference in relative knee displacement translation between the FE model and the RM. Although the DB method appears to be superior in restraining knee laxity, the margin between the two methods is not disproportionate.

The choice between SB and DB remains a controversial research topic within the orthopedic community. Towards this direction, we compared these techniques to assess their efficiency in restoring knee laxity. The comparative study is materialized by selecting a single graft with a radius of 5 mm for the SB case and two grafts of radii 2.5 mm for the DB approach. The radii were selected in an effort to achieve a comparatively equal total graft diameter in both methods. We adopted this approach based on relevant clinical studies^81,82^. In all cases, a semitendinosus graft was used. The independent variable was graft pretension prior to fixation. The results are demonstrated in Fig. 10. The best performance for the SB technique is for a graft pretension of 80 N with an absolute difference in relative knee displacement of 0.17 mm. Contrarily, the DB demonstrated the best performance for a pretension of 60N applied on both grafts. We can point out that although the DB technique appears more competent, both techniques demonstrate a comparable performance.

## Discussion

The fundamental goal of this study was to develop a computational pipeline that generates FE models to investigate the outcome of virtual ACLR surgeries. The first objective was to enable realistic subject-specific modeling to examine aspects of the surgery, such as alternative techniques, geometric graft characteristics, graft placement, material properties, and pretension. The second objective was to automate the assembly process to generate a large number of  FE models and perform sensitivity analyses. This is achieved through scripting and using open-source software tools. Finally, after validating the RM using available experiments from the Open Knee(s) project^50^, we limited our analysis to the Lachman test and “what-if” studies. The modeling process relies on MRI data to construct the subject-specific model and open-source tools for model generation, simulation, and analysis.

Starting from the surgery modeling workflow, we developed an automatic pipeline for modeling a plethora of surgery parameters directly on top of the subject’s geometries following the MRI segmentation. Regarding surgery techniques, we modeled the SB and DB ACLR methods. These are common approaches performed by surgeons and were the focus of previous FE studies^13,21,22,26,27,49^. In the scope of our work, the grafts are modeled as SB cylindrical objects with radii in the range of 1.5 to 5.5 mm. These are placed through tunnels featuring the same cylindrical shape^24,28–32,42–44,47^. The graft mesh density is higher around the tunnel insertion areas to improve the accuracy of the numerical analyses, as the developed stresses on these regions are of high interest^24,28,30,31,42–44,49^. Moreover, our workflow can be deployed to easily model approaches such as the AM and TT techniques. These are classified based on the femoral and tibial tunnel positioning^9,10,20,83–86^ (see also Sect. 3.5 of the supplementary materials). The most prominent advantage of the proposed modeling concept is that it facilitates the automatic creation of multi-featured models, suitable for ACLR FE simulations.

Regarding ligament modeling, MRI data were used to fine-tune the values of the selected constitutive model. The ligaments are modeled as nonlinear springs. In similar FE studies the ligaments were represented by volumetric meshes, and modeled as transversely isotropic hyperelastic materials explicitly taking the 3D geometry into account^25,87^. Although our modeling choice may seem as a simplification of the ligaments’ real nature, it reduces the model’s complexity while preserving a satisfying level of realism^88^. Moreover, we consider that the material properties of each ligament should be decided by conducting sensitivity analyses based on experimental data. In the case of volumetric ligament meshes, this would drastically increase the complexity of our workflow. On the contrary, permutations of the ligaments’ force - strain relationship parameters can affect knee kinematics even in dynamic activities, such as gait, leading to realistic knee movement^36^. Therefore, we decided to apply a simpler model for the ligaments and enhance its validity by performing sensitivity analyses for the parameters of interest.

Initially, the cross-section area of each ligament was estimated from the segmented geometries using PCA. By obtaining values of Young’s modulus found in the literature, we estimated the stiffness parameter of the ligaments. We compared them with reference values commonly used in other studies (Table 1). A good agreement between estimated and reference values were observed for the ACL, and MCL that is in line with other references^89–92^. Contrarily, a large discrepancy between the estimated and reference stiffness value for the PCL was observed. The underlying reason was the estimated cross-section area, which in our case was much lower. Still, the estimated area is comparable with PCL cross-section values available in related literature^91,93,94^. As for the LCL, the estimated stiffness was considerably lower than the reference value, as a side-effect of the small estimated cross-section area. However, the reference LCL cross-section area was not available to compare with. Nonetheless, similar values can be found in the literature with a consensus that LCL exhibits low cross-sectional area^95–97^. This could also betoken that structures of the PL knee should not be ignored in these calculations. Nevertheless, in the presence of high-quality MRI we can make use of subject-specific cross-section area estimates to further calibrate the model and avoid using generic reference values.

In this study, we took advantage of the laxity experiments in the Open Knee(s)^50^ project to validate the model’s response by performing sensitivity analysis on the combination of ligaments’ stiffness and prestrain values. The sensitivity analysis for the ACL revealed that a decrease in the estimated stiffness in the range of 20% and 30% is beneficial to minimize the MSE between model and experimental data. Notably, the combination of stiffness value estimated by our method and the reference prestrain factor is within the best candidate solutions with a MSE of 0.0667 mm compared to the MSE of 0.0473 mm for the best combination. Results also suggest an increase in the stiffness values of the PCL to maintain good agreement with experimental data. However, if the estimated stiffness value is used, the prestrain parameter should be increased to minimize the mismatch. The combination of initial guess parameters exhibited a MSE of 0.297 mm compared to the 0.136 mm MSE of the best solution. Overall, the combination of estimated ligament stiffness values through MRI and reference values for the prestrain give rise to responses that are close to experimental measurements from laxity experiments. Therefore, the proposed methodology could be beneficial for ACLR surgery planning when MRI data are available for both the healthy and the injured knee. We can then examine different surgeries and compare their responses with the contralateral leg^98–100^.

The Achilles heel of numerical models is the ability to validate them before performing “what-if” studies. Apart from ligament parameter validation, we examined the 6D kinematics of the knee during passive flexion and compared them with experimental data from the Open Knee(s) project. Results demonstrated an excellent match for the translational DoFs (e.g., anterior-posterior and superior-inferior) that are required for the Lachman test studies, increasing our confidence in the model. A slight discrepancy was observed for the mediolateral translation attributed to unmodeled passive tissues such as muscles or ligament bundles. In the case of rotations, we observe the same pattern for the knee adduction-abduction rotation, with our model demonstrating an increased valgus rotation for high flexion angles. However, a significant difference appears for the internal rotation. This could be attributed to the larger stiffness values of the MCL as compared to the LCL. Also, it could be due to the absence of the patellofemoral joint and the corresponding patellofemoral ligaments that is a limitation in the present study^101^. Moreover, we consider that replicating the varus - valgus and internal - external rotation tests available from the Open Knee(s) project could lead to modifications of the MCL and LCL material properties, thus further improving the remaining kinematic DoFs. Nevertheless, the model conveys accurate kinematics predictions for the directions related to the ACLR surgery and Lachman test evaluation.

The verified FE model was used as a reference to evaluate the impact of different surgical options through a FE simulation of the anterior drawer test. This allowed comparing our results with previous studies that followed the same approach^24,31,38,41–44,46,47^. Initially, we assessed the effect of graft radius in restraining relative knee displacement. Results demonstrate that increasing graft radius reduces knee instability, which is in line with other reported findings^47^. Additionally, we observed that a graft of 4 mm radius leads to satisfactory responses as predicted by the model. This graft size is a common surgery choice as reported in literature^11,102–104^. Subsequently, we performed a sensitivity analysis to investigate the impact of pretension on knee laxity. We observed that a gradual increase of graft pretension enhances knee stability, in agreement with similar studies^38,42,47^, and clinical analyses^15^. Furthermore, we observed that the minimum MSE was for a pretension of 80 N, a widely used pretension level by surgeons^15,105^.

As adjudged from these two case studies, the correlation of graft radius and pretension cannot be neglected. In this perspective, we performed a sensitivity analysis to obtain the best combination of graft radius and pretension for three graft materials. We considered that the optimal set is the most effective in reducing knee laxity as compared to the RM. As a result, the best performance in terms of minimizing relative knee displacement between simulated ACLR FE model and the RM was obtained using a gracilis graft with a radius of 2.5 mm and a pretension of 160 N. We also recorded peak values of the graft maximum principal stresses, which in almost all cases were observed around the femoral tunnel entry point similar to other studies^24,28,30,31,42–44,49^. We noticed that large pretension levels introduce very high stresses, especially for the grafts with smaller radii, with magnitudes close to failure values^106,107^. This implies that a set of parameters which may seem optimal in terms of reducing knee anterior - posterior laxity, such as the gracilis graft combination in our case, could introduce higher graft stress values with an increased potential risk of graft failure. In addition, a noticeable observation is that grafts with a radius of 4 mm and pretension of 80 demonstrate a competent performance for all tissues. The values for these two parameters are common surgery choices as stated above. The pretension value of 80 N is in the range of the maximum single hand pull that a surgeon can apply and a commonly accepted pretension value^7,108,109^. The results of our analysis indicate that the criteria for final decision on surgery parameters should not only consider the best kinematics restoration, but also the risk of potential graft failure. Hence, our framework can be applied to identify optimal graft pretension and radius for subject-specific modeling of ACLR.

Furthermore, we assessed the effect of the knee flexion angle during graft fixation on reducing knee laxity, with knee angles determined based on literature sources^24,27,38,42,43,47^. The best performance was observed for a knee angle of 20$^\circ$, albeit the difference with the knee flexion 15$^\circ$ model was minimal. Further increase in the knee flexion angle led to an upsurge of laxity. Similar optimal graft fixation angles can be found in literature^110,111^, although a commonly used fixation angle is at 30$^\circ$^112,113^. Nonetheless, the optimal knee flexion angle during graft fixation is still a controversial topic amongst clinicians. The proposed workflow could be used to investigate the most appropriate fixation angle pre-surgically taking into account subject-specific traits of the anatomical structures.

Last but not least, we performed a comparison between the SB and DB techniques. Juxtaposition studies between the two approaches remain a hot research topic since they require long-term follow-up evaluation of the reconstructed ligament^11,114,115^. In our study, the DB approach demonstrated slightly better performance in constraining relative knee displacement. The single graft with a pretension of 80 N showcased the best performance for the SB technique, while the pretension of 60 N provided the best performance for the DB. Nonetheless, the difference between the two approaches was not significant, as illustrated in Fig. 10 and derived by the presented absolute differences for both cases. Thus, our results demonstrate that the superiority of one method versus the other in restraining relative knee displacement is not clear. This is also supported by many relevant researches^116–119^. A limitation of our study is the radius choice for the two grafts in the case of DB surgery. The decision of having two grafts each of a diameter that is exactly the half of the total SB graft diameter was based on relevant clinical studies^81,82^. However, in most comparative studies between the two approaches a range of 7.5–10 mm for the SB graft radius and a range of 4–6 mm and 5–7 mm for the AM and PL DB grafts, respectively, can be found^120–122^. Although, these values seem to be proportionally close to the radii we selected in our analysis, they should not be considered insignificant when comparing the two approaches. Moreover, we should note that we did not compare the two methods in terms of restoring the rotational knee stability. Some studies support the superiority of DB approach regarding restoring knee rotational kinematics as well as minimizing risk of graft failure or reducing the chances of degenerative knee damage ^11,120^. Reduced graft failure in DB technique could be justified by the less required pretension, as in our case^120,123^. On the other hand, DB approach is arguably more complex, as it requires increased execution time and high accuracy to minimize the risk of breaking the osseous wall between the bone tunnels^11,122^.

In this study, we presented an automated workflow for modeling, simulation, and analysis of the ACLR surgery relying on subject-specific data. The pipeline can be used to assess the majority of surgery options in a simulation manner, taking advantage of open-source tools. We demonstrated the importance of extracting ligament-related modeling parameters from MRI, reinforcing the direction of subject-specific modeling. The proposed process can handily generate multiple FE models that feature different ACLR-related attributes. The response of these models is comparable to experimental data used for validation and predictions proposed by comparative studies. A limitation of our study is that we did not simulate dynamic movements to investigate long-term effects of the ACLR parameters, or conditions with increased potential risk of graft rupture and ACL surgery revision. A challenging but crucial step towards this direction is to model the patellofemoral joint structures and properly incorporate contribution from the muscles that span the knee joint^124^. These modifications would allow to investigate the role of ACL as a secondary rotation stabilizer and perform more detailed and robust comparative studies between the SB and DB surgery techniques. All these aspects can serve as future directions for further improving the current status of our work. Nonetheless, our results suggest that the presented workflow can be applied to manipulate surgery options in an effort to identify the optimal combination of ACLR parameters for a particular subject. We envision that the proposed framework can furnish a decision support tool at the disposal of surgeons and orthopaedists for ACLR surgery planning.

## Supplementary Information

Supplementary Information.

## Data Availability

The datasets generated during and/or analysed during the current study are available in the repository acl_reconstruction_data, https://simtk.org/projects/acl_surgery.
