# Supplementary material for: Evaluation of anterior cruciate ligament surgical reconstruction through finite element analysis
Source: Sci Rep. 2022 May 16;12:8044. doi: 10.1038/s41598-022-11601-1 (PMC9110399; doi:10.1038/s41598-022-11601-1)
Supplement: Supplementary file 1 — Supplementary Information. [file 41598_2022_11601_MOESM1_ESM.pdf]

Evaluation of Anterior Cruciate Ligament Surgical Reconstruction Through Finite Element Analysis

Konstantinos Risvas[1,*], Dimitar Stanev[1,2], Lefteris Benos[3], Konstantinos Filip[1], Dimitrios Tsaopoulos[3], and Konstantinos Moustakas[1]

[1]University of Patras, Department of Electrical and Computer Engineering, Patras, 25500, Greece

[2]École Polytechnique Fédérale de Lausanne, Institute of Bioengineering, Lausanne, 1015, Switzerland

[3]Institute for Bio-Economy and Agri-Technology (IBO), Centre of Research and Technology-Hellas (CERTH), 6th km Charilaou-Thermi Rd, GR 57001 Thessaloniki, Greece

[*]krisvas@ece.upatras.gr

Supplementary Material

Introduction

A workflow of the proposed study was presented in Fig. 1 of the manuscript. We made use of available MRI data to acquire subject-specific geometries of a cadaver knee. These geometries were used during the anterior cruciate ligament reconstruction (ACLR) surgery modeling step to develop different versions of a finite element (FE) knee model. The model was validated based on joint mechanics data available in the Open Knee(s) project [10]. Then, we used these models to evaluate different ACLR surgery parameters through a FE simulation of the Lachman test.

Methods

1 ACL Reconstruction Surgery Modeling

The initial step of the workflow is to import the femur and tibia geometries into the Blender software [7]. Then, two landmarks on each bone surface are selected that signify the insertion and exit points of the tunnels. The order of selection is illustrated in Fig. S1a.

The next step is to create a ACLR curve that will be manipulated to "drill" the bone tunnels and place the graft. The previously selected landmarks are the curve's control points. The curve consists of three distinct parts. The curve segments inside each bone are of order 2, and the middle part located in the space between the bones is of order 3. With this, we accomplish smoother curvature that is useful for graft placement. The linear compartments of the curve (inside the bones) permit precise

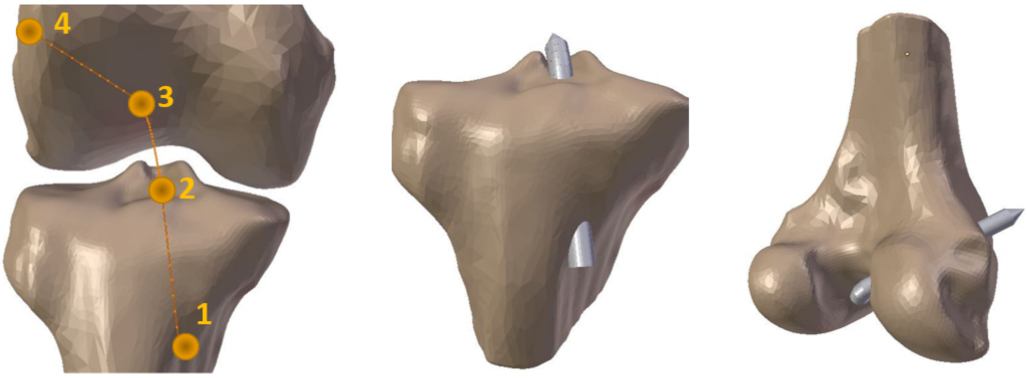

(a) **(b)** **(c)**

Figure S1: a) Four selected landmarks are used to define a NURBS Curve. On this curve, we attach the two "drills", b) Tibia “drill”, and c) Femur “drill”.

placement of the “drills” through the selected landmarks. The “drills” are cylindrical objects. Their length is slightly larger than the line segment that passes through the pair of the selected landmarks on each bone surface. By applying the Blender curve modifier to the cylindrical meshes, we can attach the “drills” to the non-uniform rational B-spline (NURBS) curve. Then, we translate them along the curve and place them in the desired positions. Finally, the tunnels are drilled utilizing the Blender’s Boolean modifier.

Also, in order to create a “crater” around the entry point of the femoral tunnel, we utilize a cone object as illustrated in Fig. S2. This eliminates acute facets on femoral surfaces and avoidance of contact breakdown during FE simulations.

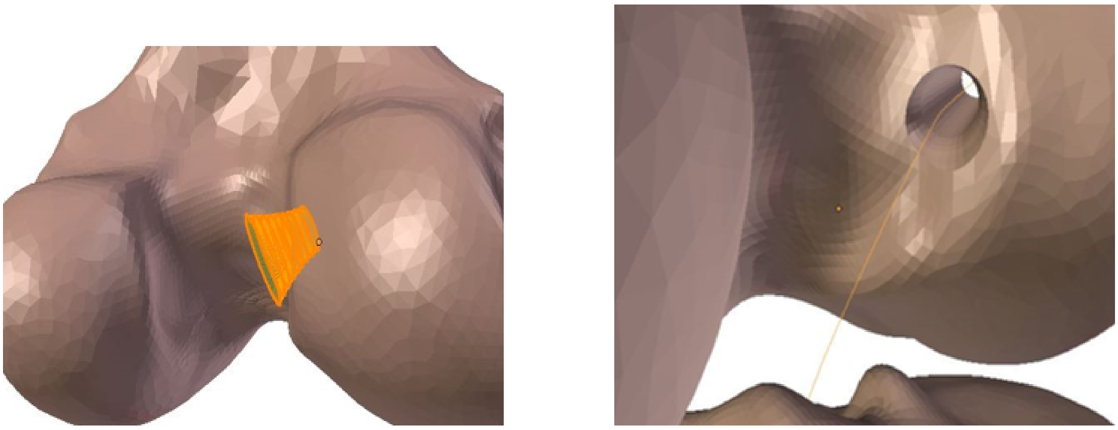

Figure S2: A cone object is used to smooth the femoral entry area.

The graft can be modeled as tetrahedral or hexahedral mesh. It is created upon a primitive grid engulfed by a circle mesh with a radius equal to that of the graft. The

nodes on the circumference of each primitive mesh are grouped to create quadrilateral faces, and thus, the base grid is generated. The edges of this mesh are then subdivided to smooth the corners and improve the mesh quality. Afterward, the edges of the refined base grid are extruded to form base cylindrical meshes consisting of brick-like elements (Fig. S3).

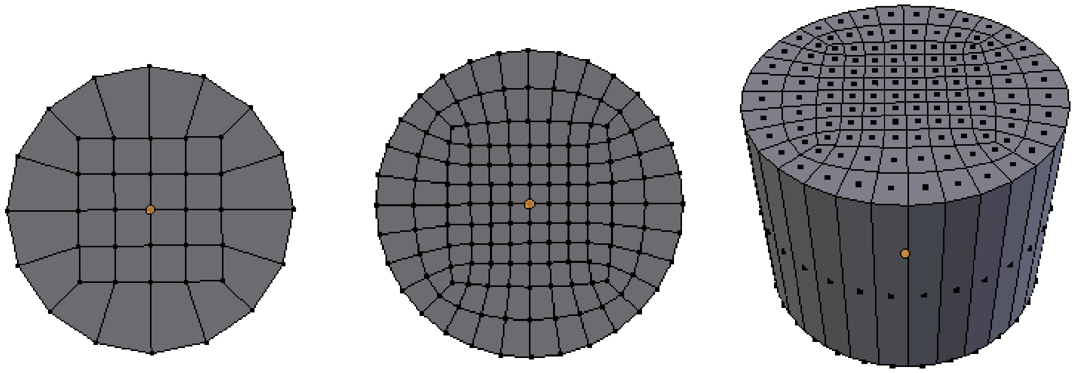

Figure S3: The graft mesh is built upon a primitive circle and grid mesh extruded to form cylindrical blocks. These are stacked together to form the final graft mesh.

These cylinders are stacked together to form the final graft mesh. The resolution and length of each cylinder can be easily modified to generate denser meshes in areas of interest to improve numerical accuracy of subsequent FE analyses (e.g., around the tunnel insertion sites). In our study, we used a denser mesh at the intermediate part of the graft with sparser segments inside the tunnels, where the graft was tied to the bone surfaces Fig. S4. Finally, the graft is attached to the NURBS curve and is placed through the tunnels.

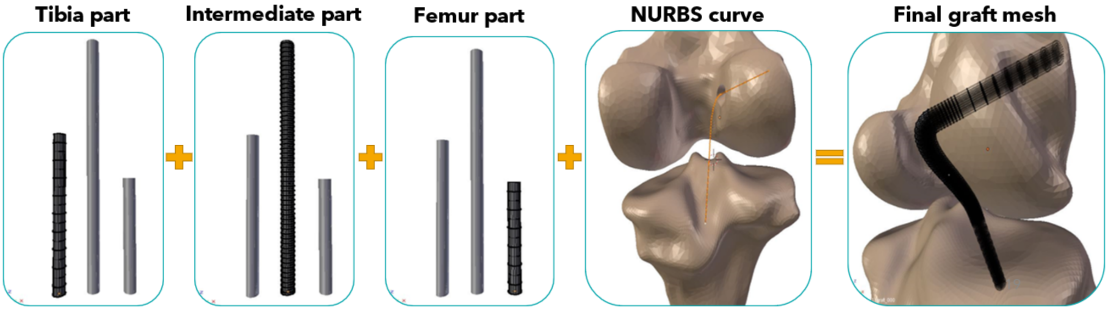

Figure S4: The cylinder blocks are stacked together to form the final graft mesh attached to the NURBS curve and placed through the tunnels. The mesh can be denser in areas of interest, as illustrated in the right-most panel.

Since Blender only supports exporting of polygon meshes (explicit surface repre-

sentation), we utilized the Blendbridge [4] software to convert the graft surface mesh to a hexahedral volumetric mesh. Additionally, the Gmsh [13] software and, in particular, the 3D Frontal unstructured algorithm was applied for graft mesh refinement. We performed a mesh quality test using the meshing quality filters of Paraview software [1]. The scaled Jacobian parameter has a value range between 0 and 1, with 1 indicating the best shape for the element. The graft mesh demonstrates values in the range of 0.79 to 1 for the scaled Jacobian. Additionally, the skewness filter was applied that also takes values between 0 and 1; but 0 indicates the best quality. The graft mesh cells demonstrated a maximum value of approximately 0.184. In general, a skewness value larger than 0.85 affects solution accuracy for hexahedral cells. Thus, the generated graft mesh exhibits good mesh quality properties. Furthermore, we store the facets of the external graft surface in the form of quartets of node indices to automatically select the contact surfaces pair in the subsequent FE simulation. It should be stressed that all the above steps are performed through scripting.

1.1 Boundary Cylinders

To achieve an automatic selection of surface pairs for contact configuration in the subsequent FE analyses, we also create cylindrical objects that enclose different parts of the graft and bones. These cylinders are also utilized for selecting specific node sets and surface parts of the graft to apply boundary conditions, such as the tension at the bottom surface of the graft.

2 Finite Element Model Development

The fundamental parts of the FE model are displayed in Fig. S5. For the sake of completeness we demonstrate a single bundle (SB) ACLR model.

2.1 Geometries

The bone geometries were acquired directly from the Open Knee(s) project and were used from the ACLR surgery modeling tool to create the reconstructed bones. The menisci and the cartilages are hexahedral meshes that were created using the knee segmentation tools provided in [22, 24]. The properties of the bone meshes are displayed in Table S1.

The mesh properties and the geometric characteristics for each graft are presented in Table S2. The SB graft mesh properties are indicative and are related to a single graft with a radius equal to 3 mm. On the other hand, for the DB technique, we have grafts with a radius of 2.5 mm.

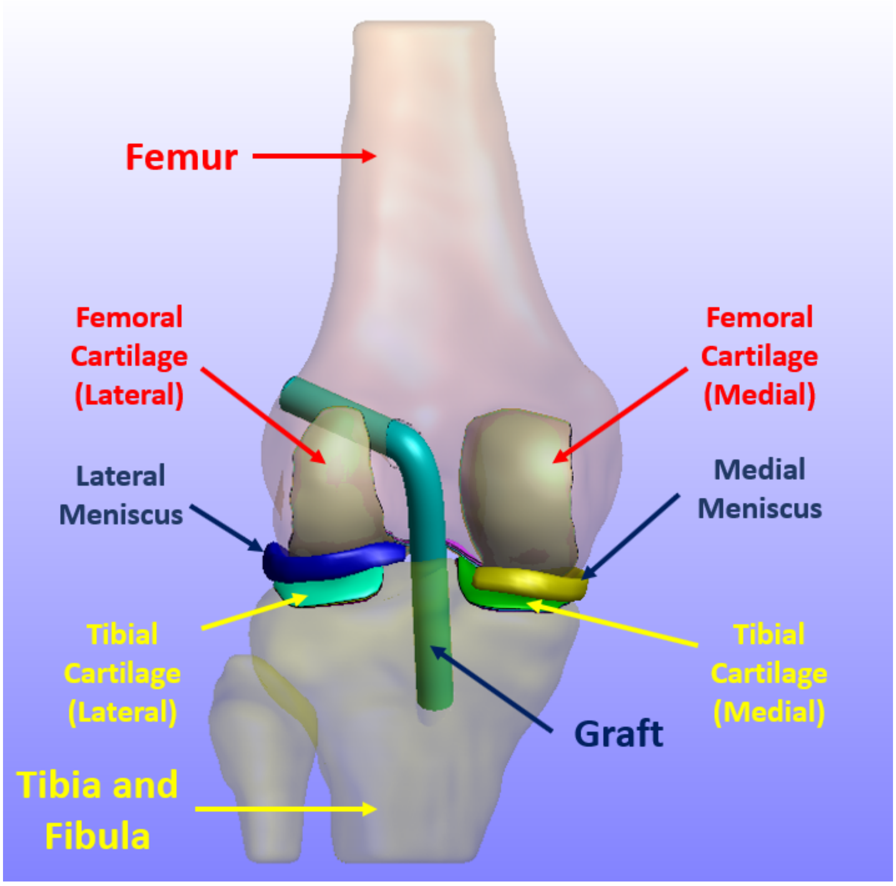

Figure S5: The parts that compromise the developed FE model. The bones are transparent to accommodate illustration of the internal anatomical structures.

Bone	Element type	Elements	Vertices
Femur	shell 3-triangular	25378	12691
Tibia	shell 3-triangular	29262	14633
Femur SB†	shell 3-triangular	45166	22583
Tibia SB†	shell 3-triangular	51652	25828
Femur DB‡	shell 3-triangular	50248	25122
Tibia DB‡	shell 3-triangular	54132	27066

† Femur and tibia for the SB technique for a graft of radius of 4mm.
‡ Femur and tibia for the double bundle (DB) technique.

Table S1: Bone mesh properties.

Graft	Element type	Elements	Vertices	Radius mm^2	Length (mm)
SB[†]	hex8	8832	10150	3	111
DB-AM[‡]	hex8	9472	10875	2.5	115
DB-PL[‡]	hex8	7168	8265	2.5	106

[†] Graft of the SB technique.
[‡] Anteromedial graft of the DB technique.
[‡] Psoterolateral graft of the DB technique.

Table S2: Graft mesh properties and geometric characteristics.

2.2 Material Properties

The femoral and tibial cartilages are modeled as three-layer geometries to represent their anatomical structure [2, 11] realistically. On each layer, a hyperelastic, uncoupled Mooney-Rivlin material is assigned. This type of material is popular for modeling the nonlinear nature of incompressible materials. The menisci are modeled using the orthotropic Fung elasticity model. The material properties of each anatomical structure are adopted by [10] and are presented in Table S3 and Table S4.

Density M/L^3	$C_1(MPa)$	$C_2(MPa)$	K
1.5e-09	0.856	0	8

Table S3: Cartilage material parameters (adopted from [10]).

Density	E_1	E_2	E_3	G_{12}	G_{23}	G_{31}	v_{12}	v_{23}	v_{31}	c	k
1.5e-09	125	27.5	27.5	2	12.5	2	0.1	0.33	0.1	1	10

Table S4: Menisci material parameters (adopted from [10]).

The grafts are modeled as transversely isotropic Mooney- Rivlin materials. The values of the model parameters are presented in Table S5 and correspond to the semitendinosus, patellar tendon, and gracilis tissues. The "fiber" parameter is a fiber distribution option. We defined a $[1,4]$ value and a "local" type. This FEBio type implies that a "local" element node numbering is applied, meaning that the orientation is determined by the numbers of the local nodes (Fig. S6). The value is selected to accommodate the fiber alignment across the longitudinal ligament axis that dictates the predominant tensile behavior of the ligaments [26].

Tissue	C_1	C_2	C_3	C_4	C_5	k	λ_m	fiber
Semitendinosus	2.75	0	0.065	115.89	512.73	1.042	103.31	[1,4]
Patellar Tendon	2.75	0	0.065	115.89	777.56	1.042	103.31	[1,4]
Gracilis	2.75	0	0.065	115.89	791.4	1.042	103.31	[1,4]

Table S5: Semitendinosus, patellar tendon, and gracilis graft material properties (adopted from [23]).

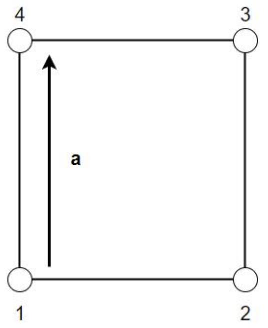

Figure S6: "Local" fiber direction to simulate the longitudinal alignment of ligament fibers.

2.3 Ligaments

The ligaments are modeled as sets of discrete elements established between two sets of vertices that lie on the bone surface meshes. These are determined from the segmented geometries and represent each ligament's origin and insertion site. The vertices of each set are sorted in ascending order based on their coordinate that is a perpendicular projection of the transverse axis of the mesh. We define one-to-one relationships between the corresponding vertices of the sorted sets. These pairs are used as the origin and attachment points for each ligament spring. The size of the smaller set determines the spring total number. To define the response of a nonlinear spring, FEBio requires the spring's force-displacement relationship. Thus, for each spring, Eq. 1 of the manuscript is adapted to estimate forces as a function of displacement $L - L_0$, where L is the length of the spring at each time step of the simulation. Moreover, we have included spring ligaments that resemble the anterolateral ligament, the posterior capsule, and the oblique arcuate ligament. Their parameters are presented in Table S6.

Ligament	Stiffness K	Reference Strain	Linearity
**ALL**	42 N	0.03	Non-Linear
**Posterior Capsule**	500 N	-0.11	Non-Linear
**Oblique Arcuate Ligament**	28 N/mm^2	-	Linear

Table S6: Stiffness parameters and reference strains for the Posterior Capsule, Arcuate and Anterolateral ligaments (adopted from [21, 15, 19, 18, 25, 9]).

3 Contacts

A step in FE modeling that cannot be ignored is the establishment of appropriate contact models. These are applied on pairs of surface sets and define their interaction. An overview of the FEBio contact models and contact pairs enacted for the FE models we used throughout this study is presented in Table S7. We also provide the reader a more intuitive representation of these contact pairs in Fig. S7.

Surface A	Surface B	Contact
Inner Femoral Cartilage	Femur	Rigid Connection
Inner Lateral Tibial Cartilage	Tibia	Rigid Connection
Inner Medial Tibial Cartilage	Tibia	Rigid Connection
Outer Femoral Cartilage	Superior Lateral Meniscus	Sliding Elastic
Outer Femoral Cartilage	Superior Medial Meniscus	Sliding Elastic
Outer Femoral Cartilage	Superior Lateral Tibial Cartilage	Sliding Elastic
Outer Femoral Cartilage	Superior Medial Tibial Cartilage	Sliding Elastic
Superior Lateral Tibial Cartilage	Inferior Lateral Meniscus	Sliding Elastic
Superior Medial Tibial Cartilage	Inferior Medial Meniscus	Sliding Elastic
Graft Surface	Femoral Tunnel	Sliding Elastic
Graft Surface	Lateral Intercondylar Notch	Sliding Elastic
Graft Surface	Tibia Tunnel and Tibial Plateau	Sliding Elastic
Top Graft Part	Femur	Tied Elastic

Table S7: Surface pairs and respective applied contact models.

3.1 Sliding Elastic Contact

The function of the sliding elastic contact is to eliminate penetration between the surfaces. It is preferred in our implementation because it performs better in simulations that involve high compression [20] which is common in the knee joint. Generally, enforcing a contact requires the definition of a master surface and a slave surface. The former is used to define the surface normal vector and tangents and to estimate the gap function.

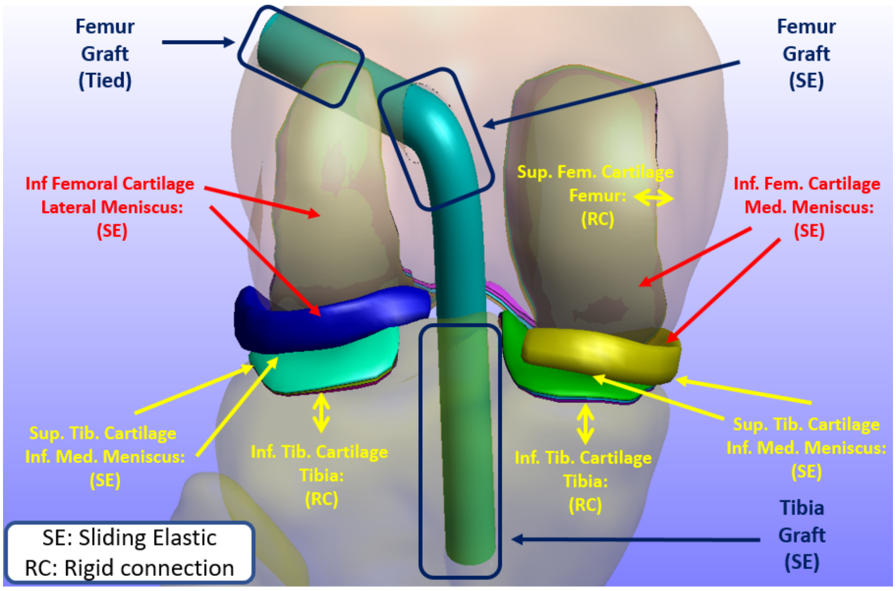

Figure S7: The established contact pairs and the respective different contact models of the developed FE model. Cartilages are connected to the femur and tibia, and a sliding contact is established between them. The same contact is established between the different surfaces of menisci and cartilages. Finally, the top part of the graft is tied to the femoral tunnel.

The latter is used to calculate the contact traction forces that prevent penetration. FEBio calculates the projection of each slave node on the master surface. This is the case of the “single-pass” method. However, the results of this method are dependent on the choice of the master and slave surfaces. To bypass this, the available “two pass” method is preferred. It combines two sequential implementations of the “single pass” approach, where the master and slave surfaces are swapped. Although this method requires more calculations, it can provide better results, especially in problems where the gap cannot be small enough because of the geometry’s curvature. Moreover, the definition of master and slave surfaces when using the “two pass” method is arbitrary. Additionally, we use the augmented Lagrangian method to approximate the Lagrangian multipliers used to calculate the contact traction forces. The augmented Lagrangian method uses the Newton-Raphson method to determine the Lagrangian multipliers based on a user-defined penalty until all constraints are satisfied. This iterative method is non-symmetric and requires extra computational power. The FEBio parameters for this contact model are specified in Table S8.

Parameter	Description	Value
**laugon**	Augmented Lagrangian method flag	1
**penalty**	Penalty of the Augmented Lagrangian method flag	0.1
**auto penalty**	auto penalty flag	1
**two pass**	"two pass" method flag	1
**min aug**	Minimum number of augmentations	0
**max aug**	Maximum number of augmentations	0
**symmetric stiffness**	Symmetric formulation	0
**search radius**	Search radius used by the algorithm to project the slave points on master facets	0.005

Table S8: Sliding elastic contact parameters (adopted from [10]).

3.2 Rigid Contacts

To define a rigid connection, a set of nodes of the deformable part and the material ID of the rigid body are required. We applied this model to fix the femoral and tibial cartilages to the femur surface and the tibial plateau, respectively.

3.3 Tied Contacts

This contact model was applied to fix the graft inside the bone tunnels. The nodes of the slave surface are tied to the faces of the master surface. A penalty factor decides the gap distance between the tied surfaces, with a higher value implying lower separation. The parameters for the tied interface are shown in Table S9. All other parameters are given their default values. This model was used as an alternative to the rigid contact described above as a workaround since activating rigid connectors during a simulation in FEBio led to convergence issues.

laugon	penalty factor	tolerance
1	1e3	0

Table S9: Tied elastic contact parameters.

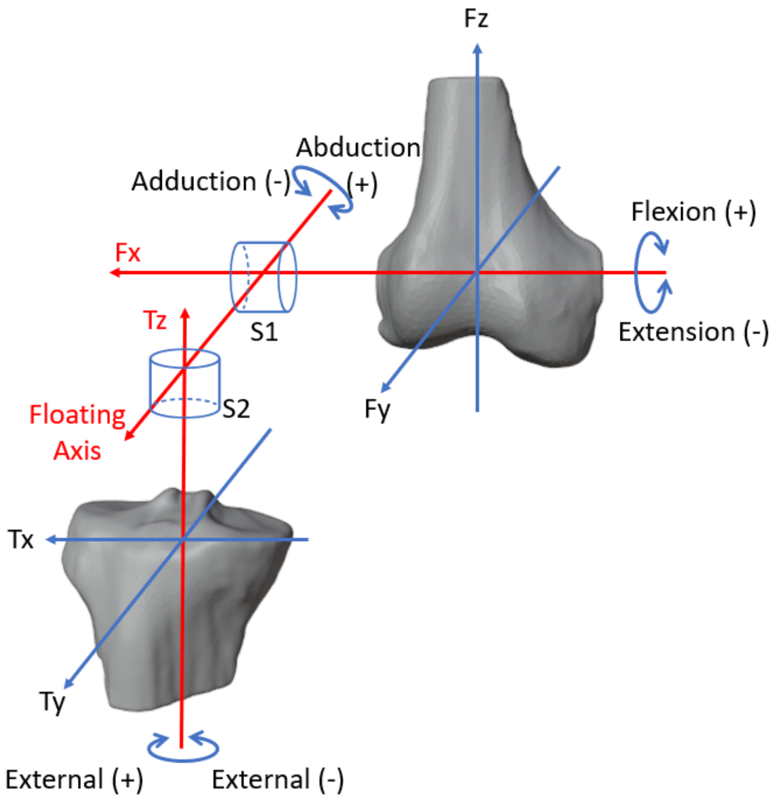

Figure S8: The knee joint coordinate system implemented in this work is based on the methodology proposed by Grood and Suntay [14]. It is composed of 4 links: the femur and tibia bones and two imaginary links s1 and s2.

3.4 Knee Joint Coordinate System

Regarding the knee joint coordinate system, we used the same convention with the Open Knee(s) project. This allowed for direct comparison between our model and the available joint mechanics data. More information can be found in the following link: Openknee_Knee_JCS. The necessary transformation matrices and registration methodology were adopted by the Open Knee(s) project repository.

An overview of the coordinate system is displayed in Fig. S8, and it was proposed by Grood and Suntay [14]. Joint kinematics are described as three red-colored axes. The first axis is the fixed femoral axis Fx, and knee flexion-extension is prescribed about it. The second is the Floating Axis, and rotation about it corresponds to knee adduction-abduction. The remaining axis is the tibial Tz axis, which is used to describe the knee internal-external rotation. The first and last links are the femur and tibia. The two intermediate links, s1 and s2, are imaginary bodies with lengths equal to zero due to the intersection between the floating and body axes.

This knee joint coordinate system is implemented in FEBio using cylindrical rigid joints. Rigid joints enforce constraints between rigid bodies and allow relative motion of the bodies only on certain degree of freedoms (DoFs). To define a FEBio cylindrical joint, a pair of rigid bodies is required, along with a common joint origin and a set of axes that decide the orientation of the allowed DoFs. These axes define two orthonormal bases e1 and e2, on each rigid body aligned at the start of the configuration. To prevent motion towards the DoFs constrained by the joint, the “force” and “moment” penalty parameters are used, which can be interpreted as stiffness parameters of linear and torsional springs respectively and produce the necessary reaction forces and moments to prevent the rigid body movement. The value of these parameters can be roughly estimated as the maximum reaction forces/moments acting on the joint divided by the gap or angular separation. The tolerance of these separations is determined by the “gaptol” and “ngtol” parameters. The reaction forces and moments are calculated using the previously described augmented Lagrangian method, where the augmentations continue until relative change in reaction forces/moments is less than the “tolerance” parameter and/or the linear gap is less than “gaptol” and/or the angular separation is less than “angtol”. We create three cylindrical joints that connect two rigid bodies (A and B) and permit one translation and one rotational DoF about the same axis to model the four-link coordinate system. The rotation of body b relative to the body a can be defined using either a prescribed rotation or a prescribed moment about the joint axis, but not simultaneously both. Similarly, translation of body b relative to the body a or a force acting along the joint axis can be prescribed. Again these elements cannot be used at once. Also, two dummy rigid bodies named “femur dummy” and “tibia dummy” with the same material parameters as femur and tibia are created to represent the imaginary links.

In Table S10, the parameters of the three cylindrical connectors that comprise the tibiofemoral joint are presented.

**Parameter**	**Medial-Lateral**	**Anterior-Posterior**	**Superior-Inferior**
Rigid body A	femur dummy	femur dummy	tibia dummy
Rigid body B	femur	tibia dummy	tibia
Tolerance	0	0	0
gaptol	0.01	0.01	0.01
angtol	0.01	0.01	0.01
Force penalty	1e04	1e04	1e04
Moment penalty	1e05	1e05	1e05
Joint origin	(-3.98,3.58,-11.27)	(-2.33,3.52,-11.38)	(-5.48,-5.36,-37.85)
Joint axis	(0.99,-0.04,-0.07)	(0.04,0.99,-0.08)	(0.11,0.08,0.99)

Table S10: Rigid joint parameters (adopted from [10]).

Simulation Parameters

Before advancing with the individual simulation scenarios, we describe the parameters defined in FEBio for controlling the simulation progress in a Structural Mechanics analysis mode. Most of the implemented parameters are the FEBio defaults. The *dtol* and *symmetric stiffness* parameters should be defined for a structural analysis. The *symmetric stiffness* value is always equal to zero to enable a nonlinear formulation and to accommodate the inherent nonlinear nature of the enforced contacts and rigid joint constraints. An overall description is displayed in Table S11. The value of *dtol* decides the convergence displacement tolerance. An overall description is displayed in Table S11.

Parameter	Description	Value
**Step size**	Initial time step size	1e-5
**Time steps**	Total number of time steps	Step running time/ Step size
**Min step size**	Minimum time step size	0.0
**Max step size**	Maximum time step size	0.05
**Auto time stepper**	Auto-adjusts the time step	Enabled
**Use must points**	Define points where FEBio must pass through	Enabled
**Max retries**	Maximum number of retries per time step	30
**Optimal iterations**	Optimal number of iterations	10
**Symmetric Stiffness**	Symmetric formulation	0
**Displacement tolerance**	Convergence displacement tolerance	0.01
**Energy tolerance**	Convergence energy tolerance	0.1
**Quasi-Newton method**	Quasi-Newton update method	BROYDEN
**Line search tolerance**	Line search method to improve the convergence of nonlinear Newton solution algorithm	0.9
**Max reformations**	Max number of stiffness reformations	25
**Max updates**	Max number of stiffness updates	10
**Diverge reform**	Option for reforming stiffness matrix when solution diverges	Enabled

Table S11: Control parameters.

The fundamental steps of the Lachman simulation pipeline are presented in Fig. S9. Initially, the knee is flexed up to the desired fixation angle. Then, the tensile load is applied as a pressure load distributed on the graft bottom surface equal to the desired tension force divided by the graft cross-section area. Finally, the graft top part is tied

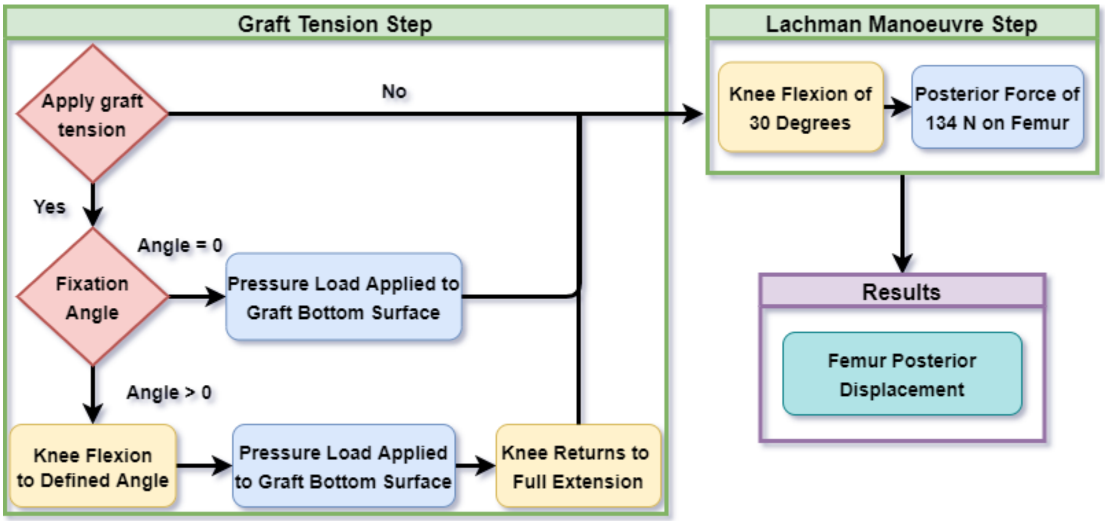

Figure S9: Overview of the Lachman test simulation flow. The containers correspond to the simulation steps. Each of them contains rounded rectangles that signal different stages of the simulation step. We highlight spans of the same step where knee flexion-extension is prescribed with yellow color. With light blue, we denote periods where loading is applied. Finally, with ciel color, we present the results that are generated in the post-processing step. In the healthy knee model, the simulation includes only the "Lachman manouevre" step. On the other hand, when the SB and DB models are assessed, the "Graft pretension" step is performed to apply an initial tension to the graft and fix it through the tunnels under a selected fixation angle (Diagrams.net, v17.2.1, `https://www.diagrams.net/`).

to the femur. After the tension step, the graft bottom surface is fixed in all DoFs, and the knee returns to the default full extension pose. Consequently, the simulation of the Lachman test inaugurates as described by the respective block. The knee is flexed at 30 degrees, and a posterior force is applied to the origin of the femoral coordinate system. The tibia is fixed throughout all Lachman simulations, whereas the femur is now fixed only for the internal-external rotational DoF. Since we focus exclusively on studying the relative displacement along the anterior/posterior direction, the role of anterior cruciate ligament (ACL) as a secondary knee rotation stabilizer is not considered. At the end of the simulation, the induced knee relative displacement and graft stresses are retrieved.

Results

3.5 ACLR Surgery Modeling

Here we present some results that highlight the potential of the ACLR surgery modeling workflow in modeling different surgery techniques. In Fig. S10, we demonstrate cases that we have investigated for comparing the SB anteromedial (AM) and transtibial (TT) portal techniques.

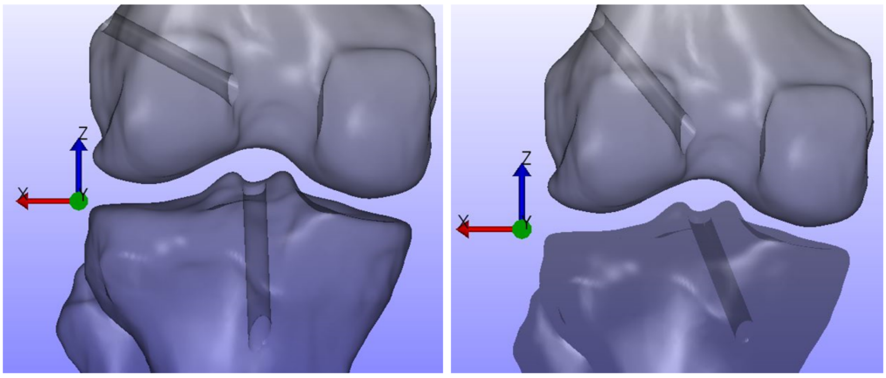

Figure S10: AM (left) and TT (right) portal techniques modeled using the proposed workflow.

These were exploited at an initial stage to assess stresses developed in the femoral insertion area, with the AM technique demonstrating higher stress values due to increased graft bending angle. However, the advantage of the AM technique is the placement of the femoral tunnel close to the native ACL footprint, and the better kinematics restoration [12, 17, 6]. For this, we have started working with dynamic movements, where the model's behavior and the surgery parameters' influence can be further investigated. Furthermore, we have worked with graft models that consist of multiple bundles. The objective is to evaluate different stresses on each bundle as multi-bundle grafts are broadly used by surgeons [8, 5, 16].

3.6 Estimation of Ligament Stiffness Through MRI

We demonstrated in the main manuscript a method for estimating each ligament stiffness exploiting the segmented geometries. In Table S12, we present the mean stiffness values for five subjects of the Open Knee(s) project that had the demonstrated geometries with the highest mesh quality. We observe that for these subjects the ACL and medial collateral ligament (MCL) mean estimated stiffness are close to the reference values. The posterior cruciate ligament (PCL) mean stiffness value is relatively smaller than the respective reference value. Finally, we observe a large difference between stiffness values for the lateral collateral ligament (LCL) ligament. Although the method we

propose is sensitive to the quality of the available segmented geometries, it provides a good estimation of ligament stiffness considering the subject-specific characteristics of these structures.

**Ligament**	**Young's modulus** (MPa)	**PCA**† **Area (mm)**	**Reference**‡ **Area (mm)**	**PCA Stiffness (N)**	**Reference Stiffness (N)**
**ACL**	355	29.25	35	10091	10000
**PCL**	304	49.87	60	17206	20100
**MCL**	355	20.74	24	7156	8250
**LCL**	355	6.41	N/A	2213	6000

† Mean area estimated from the geometries of five Open Knee(s) subjects [10].
‡ As "reference" we denote the values that are acquired from [3].

Table S12: Ligament parameter values after principal component analysis (PCA) analysis. The stiffness values are estimated as the product of the cross-section area and Young's modulus. In our study, we acquired similar values with the Blakenvoort values for the ACL and MCL ligaments.

Bibliography

[1] J. Ahrens, Berk Geveci, and C. Law. Paraview: An end-user tool for large-data visualization. In *The Visualization Handbook*, 2005.

[2] Abhijit Bhosale and James Richardson. Articular cartilage: Structure, injuries and review of management. *British medical bulletin*, 87:77–95, 09 2008.

[3] Leendert Blankevoort, Jan Kuiper, R Huiskes, and H.J. Grootenboer. Articular contact in a three-dimensional model of the knee. *Journal of biomechanics*, 24:1019–31, 02 1991.

[4] Bollenbach, Garry. https://blenbridge.sourceforge.io, 2018.

[5] Giuliano Cerulli, Giacomo Placella, Enrico Sebastiani, Meshack Tei, Andrea Speziali, and Francesco Manfreda. Acl reconstruction: Choosing the graft. *Joints*, 1:18–24, 03 2013.

[6] Haitao Chen, Kai Tie, Yongjian Qi, Bin Li, Biao Chen, and Liaobin Chen. Anteromedial versus transtibial technique in single-bundle autologous hamstring acl reconstruction: A meta-analysis of prospective randomized controlled trials. *Journal of Orthopaedic Surgery and Research*, 12, 12 2017.

[7] Blender Online Community. *Blender - a 3D modelling and rendering package*. Blender Foundation, Stichting Blender Foundation, Amsterdam, 2018.

[8] Ish Dhammi, Rehan-Ul Haq, and Sudhir Kumar. Graft choices for anterior cruciate ligament reconstruction. *Indian journal of orthopaedics*, 49:127–8, 05 2015.

[9] Shriram Duraisamy, Go Yamako, Etsuo Chosa, Lee Dave, and Karupppasamy Subburaj. Effects of a valgus unloader brace in the medial meniscectomized knee joint: a biomechanical study. *Journal of Orthopaedic Surgery and Research*, 14:1–13, 02 2019.

[10] Ahmet Erdemir. Open knee: Open source modeling and simulation in knee biomechanics. *The journal of knee surgery*, 29, 10 2015.

[11] Alice Fox, Asheesh Bedi, and Scott Rodeo. The basic science of articular cartilage: Structure, composition, and function. *Sports health*, 1:461–8, 11 2009.

[12] Hemanth Gadikota, Jae Ang Sim, Ali Hosseini, Thomas Gill, and Guoan Li. The relationship between femoral tunnels created by the transtibial, anteromedial portal, and outside-in techniques and the anterior cruciate ligament footprint. *The American journal of sports medicine*, 40:882–8, 02 2012.

[13] Christophe Geuzaine and Jean-François Remacle. Gmsh: A 3-D finite element mesh generator with built-in pre- and post-processing facilities. *International Journal for Numerical Methods in Engineering*, 79(11):1309–1331, September 2009.

[14] Edward Grood and W.J. Suntay. A joint coordinate system for the clinical description of three-dimensional motions: Application to the knee. *Journal of biomechanical engineering*, 105:136–44, 06 1983.

[15] Hideya Ishigooka, Toshihiro Sugihara, Kuniaki Shimizu, Haruhito Aoki, and Kazuaki Hirata. Anatomical study of the popliteofibular ligament and surrounding structures. *Journal of Orthopaedic Science*, 9(1):51–58, 2004.

[16] Christopher Kaeding, Kurt Spindler, Laura Huston, and Alex Zajichek. Acl reconstruction in high school and college-aged athletes: Does autograft choice affect recurrent acl revision rates? *Orthopaedic Journal of Sports Medicine*, 7:2325967119S0028, 07 2019.

[17] Bekir Kilinc, Adnan Kara, Yunus Oc, Haluk Celik, Savas Camur, Emre Bilgin, Yunus Erten, Türker Sahinkaya, and Osman Eren. Transtibial vs anatomical single bundle technique for anterior cruciate ligament reconstruction: A retrospective cohort study. *International Journal of Surgery (London, England)*, 29:62–69, 03 2016.

[18] Robert LaPrade, Patrick Morgan, Fred Wentorf, Steinar Johansen, and Lars Engebretsen. The anatomy of the posterior aspect of the knee: An anatomic study. *The Journal of Bone and Joint Surgery-American Volume*, 89:758–764, 04 2007.

[19] Robert F. LaPrade, Andy Tso, and Fred A. Wentorf. Force measurements on the fibular collateral ligament, popliteofibular ligament, and popliteus tendon to applied loads. *The American Journal of Sports Medicine*, 32(7):1695–1701, 2004. PMID: 15494335.

[20] Steve Maas, Benjamin Ellis, Gerard Ateshian, and Jeffrey Weiss. Febio: Finite elements for biomechanics. *Journal of biomechanical engineering*, 134:011005, 01 2012.

[21] Hamid Naghibi, Dennis Janssen, Tony Tienen, Sebastiaan Groes, Ton Van den Boogaard, and Nico Verdonschot. A novel approach for optimal graft positioning and tensioning in anterior cruciate ligament reconstructive surgery based on the finite element modeling technique. *The Knee*, 02 2020.

[22] Filippos Nikolopoulos, Dimitar Stanev, and Konstantinos Moustakas. Personalized knee geometry modeling based on multi-atlas segmentation and mesh refinement. *IEEE Access*, 8:56766–56781, 03 2020.

[23] Estefania Peña, Miguel Martínez, Begoña Calvo, Daniel Palanca, and Manuel doblaré. A finite element simulation of the effect of graft stiffness and graft tensioning in acl reconstruction. *Clinical biomechanics (Bristol, Avon)*, 20:636–44, 08 2005.

[24] B. Rodriguez-Vila, P. Sánchez-González, I. Oropesa, E. J. Gomez, and D. M. Pierce. Automated hexahedral meshing of knee cartilage structures – application to data from the osteoarthritis initiative. *Computer Methods in Biomechanics and Biomedical Engineering*, 20(14):1543–1553, 2017. PMID: 29017357.

[25] Takehiko Sugita and Andrew A. Amis. Anatomic and biomechanical study of the lateral collateral and popliteofibular ligaments. *The American Journal of Sports Medicine*, 29(4):466–472, 2001. PMID: 11476388.

[26] Jeffrey Weiss, John Gardiner, Benjamin Ellis, Trevor Lujan, and Nikhil Phatak. Three-dimensional finite element modeling of ligaments: Technical aspects. *Medical engineering and physics*, 27:845–61, 01 2006.
